# Acquired thrombotic thrombocytopenic purpura with isolated *CFHR3/1* deletion—rapid remission following complement blockade

**DOI:** 10.1007/s00467-018-3957-8

**Published:** 2018-05-04

**Authors:** Martin Bitzan, Rawan M. Hammad, Arnaud Bonnefoy, Watfa Shahwan Al Dhaheri, Catherine Vézina, Georges-Étienne Rivard

**Affiliations:** 1 Division of Nephrology, Department of Pediatrics, Montreal Children’s Hospital, McGill University Health Centre, Room B RC.6651, Montreal, Québec H4A 3J1 Canada; 2 Service d’hématologie-oncologie, CHU Sainte-Justine and Université de Montréal, Montréal, Canada; 3 Present Address: Department of Pediatric, Tawam Hospital, Al Ain, United Arab Emirates; 4 Division of Hematology/Oncology, Department of Pediatrics, Montreal Children’s Hospital, McGill University Health Centre, Montreal, Canada

**Keywords:** ADAMTS13, Atypical hemolytic uremic syndrome, Complement factor H-related protein, Eculizumab, Thrombotic microangiopathy, Ultra-large von Willebrand factor multimers

## Abstract

**Background:**

Thrombotic thrombocytopenic purpura (TTP) is caused by the abundance of uncleaved ultralarge von Willebrand factor multimers (ULvWF) due to acquired (autoantibody-mediated) or congenital vWF protease ADAMTS13 deficiency. Current treatment recommendations include plasma exchange therapy and immunosuppression for the acquired form and (fresh) frozen plasma for congenital TTP.

**Case-diagnosis/treatment:**

A previously healthy, 3-year-old boy presented with acute microangiopathic hemolytic anemia, thrombocytopenia, erythrocyturia and mild proteinuria, but normal renal function, and elevated circulating sC5b-9 levels indicating complement activation. He was diagnosed with atypical hemolytic uremic syndrome and treated with a single dose of eculizumab, followed by prompt resolution of all hematological parameters. However, undetectably low plasma ADAMTS13 activity in the pre-treatment sample, associated with inhibitory ADAMTS13 antibodies, subsequently changed the diagnosis to acquired TTP. vWF protease activity normalized within 15 months without further treatment, and the patient remained in long-term clinical and laboratory remission. Extensive laboratory workup revealed a homozygous deletion of CFHR3/1 negative for anti-CFH antibodies, but no mutations of ADAMTS13, (other) alternative pathway of complement regulators or coagulation factors.

**Conclusions:**

This case, together with a previous report of a boy with congenital TTP (Pecoraro et al. Am J Kidney Dis 66:1067, 2015), strengthens evolving in-vitro and ex-vivo evidence that ULvWF interferes with complement regulation and contributes to the TTP phenotype. Comprehensive, prospective complement studies in patients with TTP may lead to a better pathophysiological understanding and novel treatment approaches for acquired or congenital forms.

## Introduction

Thrombotic microangiopathies (TMA) comprise a heterogeneous group of hereditary and acquired disorders characterized by microangiopathic hemolytic anemia, thrombocytopenia, and organ damage associated with capillary and arteriolar thrombosis and, in many instances, vessel wall abnormalities [1]. Thrombotic thrombocytopenic purpura (TTP) results from deficient von Willebrand factor (vWF) cleaving zinc metalloprotease ADAMTS13 [2]. vWF is released from Weibel-Palade bodies in vascular endothelial cells and platelet alpha granules and participates in platelet adhesion and aggregation especially in conditions of high-shear stress [3]. ADAMTS13 controls the hemostatic function of endothelial cell- and platelet-derived vWF by cleaving hyper adhesive ultra-large vWF multimers (ULvWF) [2,3]. Abundance of uncleaved ULvWF multimers leads to occlusive microthrombi in small blood vessels [1.^3^]. TTP can be congenital, due to homozygous or compound heterozygous mutations of *ADAMTS13* (Upshaw-Schulman syndrome) or acquired (aTTP) [4]. The latter is caused by inhibitory ADAMTS13 antibodies [2,4]. Diminished metalloprotease activity below 10% of normal differentiates TTP from other forms of TMA [1,4]. Intensive plasma exchange (PLEX) therapy combined with immunosuppression has improved the previously dismal outcome of aTTP [2,4].

Complement-mediated haemolytic uremic syndrome (HUS), commonly referred to as “atypical” (aHUS), is caused by mutations of genes encoding regulatory proteins of the alternative pathway of complement (APC) or the coagulation cascade [5]. Ten to 20% of cases are due to inhibitory autoantibodies, predominantly against complement factor H (CFH), associated with biallelic deletions of *CFHR3/1* (“DEAP HUS,” deficiency of CFHR proteins and CFH autoantibody positive) [6]. Uncontrolled APC activation results in the formation of membrane attack complex (MAC), endothelial injury, and a prothrombotic phenotype. However, the etiological diagnosis of TMA can be challenging, particularly where diagnostic assays are not readily available. aHUS is effectively treated with the anti-C5 antibody eculizumab [7,8], while PLEX and immunosuppression are recommended for patients with autoimmune (DEAP) HUS [7], similar to aTTP [2,4].

Here we present a 3-year-old boy with an eventual diagnosis of aTTP who recovered promptly after a single dose of eculizumab. The case challenges the accepted diagnostic and therapeutic dichotomy between (atypical) HUS and TTP.

## Case report

A previously healthy 3-year-old Moroccan boy was admitted with anemia and thrombocytopenia. He had been well until 3 weeks prior to presentation, when he developed a febrile erythematous rash. Fever recurred a week before admission, associated with lethargy, vomiting, and non-bloody diarrhea. Family history is negative for kidney or hematological disorders; the non-consanguineous parents and the boy’s three siblings are healthy.

The patient appeared pale, with bruises on abdomen, back, and lower extremities. The clinical exam was otherwise unremarkable. Laboratory work-up revealed hemolytic anemia with marked reticulocytosis, presence of schistocytes, profound thrombocytopenia, elevated uric acid, and normal serum creatinine concentrations. Plasma haptoglobin was undetectable, lactate dehydrogenase (LDH) elevated, and direct Coombs test negative. A stool sample was negative for *E. coli* O157:H7. Anti-streptolysin titers were only marginally elevated. D-dimers were increased to 2.49 μg/mL fibrinogen-equivalent units (N 0.02–0.47 μg/mL). Prothrombin, international normalized ratio (INR), partial thromboplastin time, fibrinogen, and C3 and C4 concentrations were normal, and sC5b-9 was increased to 653 ng/mL (normal < 300 ng/mL; SC5b-9 Plus MicroVue, ELISA, TECOmedical/Quidel, San Diego, CA). Urinalysis revealed microscopic erythrocyturia and mild proteinuria. On Day 2, the patient received transfusions of red blood cells and platelets. Hemoglobin (Hb) continued to fall to 48 g/L, and platelets dropped to 5 × 10^9^/L within 2 days of the transfusions (Table 1).

**Table 1:** Biological parameters pre- and post-anti-C5 antibody infusion^a^

Parameter ^*a*^	Reference range	Presentation	Peak/nadir	Pre-eculizumab	Resolution ^b^	Last measurement
Hemoglobin	105–135 g/L	60 (D − 1)	48 (D − 1)	74 ^c^	108 (D + 17)	125 (D + 1494)
Serum lactate dehydrogenase (LDH)	142–297 U/L	1205 (D − 1)	1205 (D − 1)	926	285 (D + 17)	211 (D + 1494)
Haptoglobin	0.69–1.96 g/L	< 0.06 (D − 1)	< 0.06 (D − 1/+17)	< 0.06	0.36/0.89 (D + 59/82)	0.83 (D + 1494) ^d^
Reticulocytes	0.002–0.020 × 10^9^/L	NA	0.116 (D − 0)	0.116	0.018 (D + 12/17)	0.012 (D + 521)
Platelet count	140–450 × 10^9^/L	8 (D − 1)	5 (D + 1)	21 ^c^	202 (D + 17)	311 (D + 1494)
eGFR ^e^	mL/min/1.73m^2^	110 (D − 1)	110 (D − 1)	113	139 (D + 10)	122 (D + 1301)
ADAMTS13^f^	56–133%	NA	< 10 (D − 0/+ 17)	< 10	< 10/47 (D + 17/82) ^f^	> 150 (D + 1301)
Anti-ADAMTS13	Negative	NA	1:64 (D − 0)	1:64	1:4/negative (D + 360/475)	negative (D + 942/1304)
C3	0.75–1.40 g/L	NA	0.85 (D − 0)	0.85	NA	1.37 (D + 1494)
C4	0.17–0.47 g/L	NA	0.35 (D − 0)	0.35	NA	0.39 (D + 1494)
CH50	69–129%	87 (D − 0)	< 1 (D + 1) ^g^	87	9/79 (D + 31/59) ^g^	103 (D + 528)
AH50	30–113%	42 (D − 0)	< 1 (D + 1) ^g^	42	29/76 (D + 59/82) ^g^	78 (D + 528)
sC5b-9	< 300 ng/mL	NA	653 ^h^ (D − 0)	653	262 (D + 81/360)	106 (D + 1301) ^h^
Anti-CFH antibody	Negative	Negative	Negative	Negative	Negative	Negative
Urine protein (dipstick)	Negative (g/L)	0.3 (D − 1)	0.3 (D − 1)	NA	Neg (D + 1)	Neg (D + 1301)
Urine blood/hemoglobin (dipstick)	Negative	Moderate (D − 1)	Moderate (D − 1/+ 2)	NA	Small/negative (D + 5/6)	negative (+ 1301)
U protein/creatinine	< 0.020 g/mmol	NA	0.050 (D + 5)	NA	0.021 (D + 6)	0.012 (D + 1301)
Urine RBC (microscopy)	Per HPF	25–30 (D − 0)	25–30 (D − 0)	25–30	Negative (D + 6)	Negative (D + 1301)

*D* day (relative to eculizumab infusion), *eGFR* estimated glomerular filtration rate, *HPF* high-power field, *NA* not available/not applicable, *RBC* red blood cells

^a^See text for additional laboratory results. “D” (day) refers to the number of days before or after eculizumab infusion (“D 0” indicates the day of infusion, “D − 0” immediately prior to infusion)

^b^Resolution documented on the second of the two indicated days (where interim measurements were not obtained)

^c^After transfusion of packed red blood cells and platelets

^d^Haptoglobin peaked after recovery at 2.89 (D + 523)

^e^Schwartz (CKiD) formula

^f^Fluorescence resonance energy transfer (FRETS-VWF73) assay

^g^Eculizumab-induced complement blockade

**Fig. 1:**
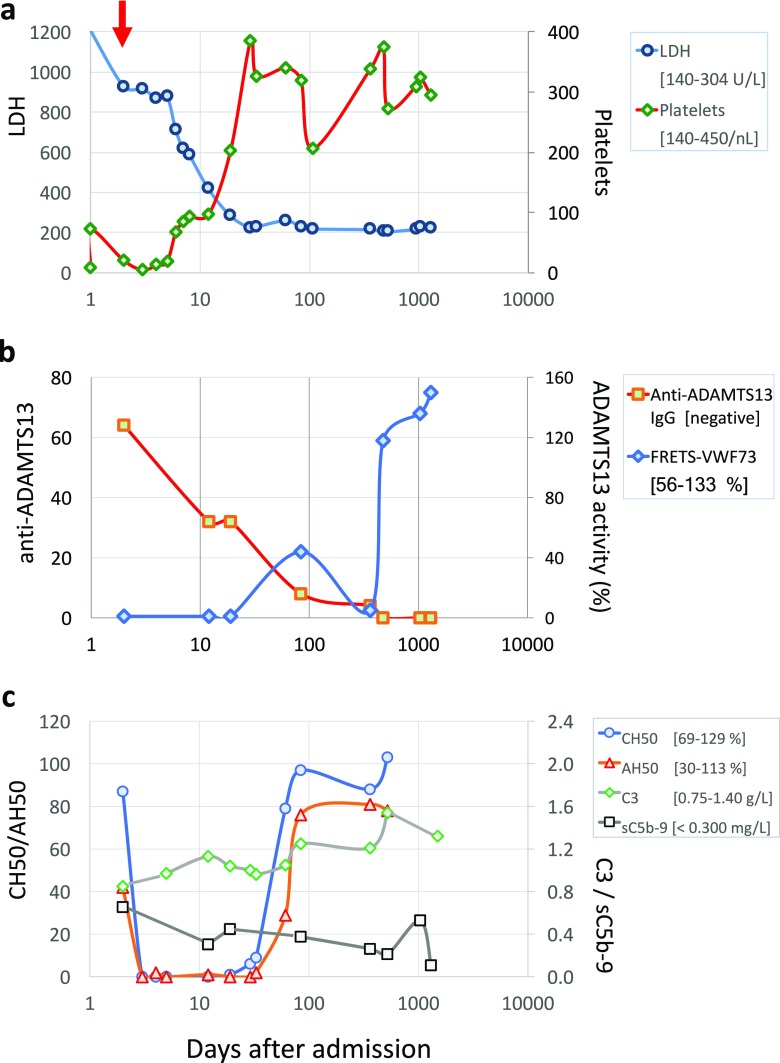
Disease course **a** Platelet count and lactate dehydrogenase (LDH) levels pre- and post-therapy. Eculizumab was given on Day 2 of admission (red arrow). The platelet count increased steadily from 5 × 10^9^/L on Day 3 and normalized 17 days after the antibody infusion, similar to LDH and hemoglobin (108 g/L on day 17 of eculizumab therapy; not shown). **b** ADAMTS13 activity and anti-ADAMTS13 antibodies following eculizumab infusion. **c** Results of global classical (CH50) and alternative pathway activities (AH50), C3 and soluble MAC (sC5b-9) concentrations during acute disease and long-term follow-up

^h^Intermittent rise of plasma sC5b-9 concentration to 530 ng/mL was incidentally detected more than 2 ½ years after initial presentation (see Fig. 1 and Discussion)

A tentative diagnosis of aHUS was made, and a single dose of eculizumab (~ 900 mg/m^2^) was given 2 days after admission. The patient was vaccinated against *N. meningitidis* and started amoxicillin prophylaxis. Platelet count, Hb, and LDH started to improve after 4 days and normalized within 17 days. The diagnosis was corrected to TTP several days after discharge from hospital, when the ADAMTS13 activity in the pre-treatment plasma sample was found to be unmeasurably low using the fluorescence resonance energy transfer (FRETS-VWF73 substrate) assay (Peptide International Inc. Louisville KY) [9]. The patient also had anti-ADAMTS13 IgG antibodies (1:64) (in-house titration ELISA with recombinant ADAMTS-13 (Baxter, Mississauga, Canada) as target antigen and serial plasma dilutions). Incubating patient and reference plasma (ADAMTS13 activity 0 and 100%, respectively) in equal volumes at 37 °C for 30 min reduced the ADAMTS13 activity in the mixture to 0%, indicating the presence of an inhibitor in an assay analogous to the Bethesda assay using the FRETS-VWF73 substrate. Due to rapid clinical and laboratory improvement following treatment with eculizumab, we refrained from PLEX and immunosuppressive therapy. ADAMTS13 activity normalized completely after 15 months (Fig. 1 and Table 1).

The initial mutation screen for *ADAMTS13* and genes encoding complement factors CFH, CFI, and CFB, CD46/membrane cofactor protein (MCP), factor H-related protein (CFHR) 5, C3, apelin and thrombomodulin was negative. CFH protein concentration was normal. Comprehensive re-testing confirmed the previous results and excluded mutations of diacylglycerol kinase-epsilon (*DGKE*), plasminogen (*PLG*) and methylmalonic aciduria and homocystinuria, cblC complementation type (*MMACHC*), but identified a homozygous deletion of *CFHR3/1*. No anti-CFH antibodies were demonstrated during active disease and follow-up, and there was no documented relapse of TTP or TMA over the 4 years of observation. A moderate, temporary increase of plasmatic sC5b-9 was noted > 2½ years after presentation in the absence of clinical symptoms or hematological evidence of TMA. At the time, ADAMTS13 activity had normalized, and anti-CFH antibodies were undetectable (Table 1 and Fig. 1).

## Discussion

We report a young boy with acquired TTP, with non-measureable ADAMTS13 activity and elevated, inhibitory anti-ADAMTS13 antibodies. He had no neurological signs, and apart from erythrocyturia and mild proteinuria, renal function remained normal throughout the disease course. The case is noteworthy because of documented complement activation and swift clinical and laboratory resolution following treatment with eculizumab, without PLEX or immunosuppressive therapy.

The differentiation between TTP and aHUS can be challenging [10–12]. Our patient was found to have a homozygous deletion of *CFHR3/1* without detectable anti-CFH antibodies. Deletions in the *CHFR3/1* locus have been noted in 5–8% of the healthy population, with wide ethnic diversity [13], and are believed to be of no consequence in the absence of anti-CFH antibodies [6,14]. At present, a link between *CFHR* deletion and TTP has not been formally established. However, it has been speculated that lack of CFHR expression predisposes to autoimmune diseases [15]. Interestingly, we documented retrospectively a second “silent” rise of plasma sC5b-9 concentrations long after the resolution of TTP, without apparent hemolysis and thrombocytopenia and in the absence of anti-CFH autoantibodies (Fig. 1). We posit that intermittent or abortive episodes of APC activation and sMAC formation occur in predisposed individuals, depending on stimulus type and intensity [16]. While increased levels of sC5b-9 indicate that the terminal complement cascade has been activated, we do not know what triggered this biological event 2 ½ years after the initial presentation, and the current literature does not provide an answer concerning the frequency and specificity of increased sC5b-9 measurements or what constitutes an indication for treatment. By way of analogy, Page et al. [17] recently reported that TTP patients experience clinically uneventful episodes of diminished ADAMTS13 activity < 10% during periods of disease remission. Comprehensive and prospective, functional studies are needed to better understand the biological and clinical importance of these observations. Likewise, the temporary increase of ADAMTS13 (FRETS-VWF73) activity on day 84 of admission, confirmed in repeat assays, was unexpected in view of still detectable anti-ADAMTS13 antibodies and remains unexplained. In contrast, the documented normalization of FRETS-VWF73 assay results 15 months after disease onset correlated well with the disappearance of anti-ADAMTS13 antibodies (Fig. 1, panel B).

There is evolving experimental and ex vivo evidence of complement activation and dysregulation in TTP. For example, Turner and Moake [18] described the assembly and activation of complement by endothelial cell-anchored ULvWF molecules in vitro. Reti et al. demonstrated increased plasma concentrations of sC5b-9 and C3a in a series of patients with aTTP [19]. Plasma from TTP patients was found to contain significantly higher levels of complement-coated endothelial microparticles than controls [20]. The latter authors also showed C3 deposition on vWF-platelet strings and primary glomerular endothelial cells exposed to plasma from TTP patients in vitro, under shear. These and other studies [21–23] suggested that cleaved vWF serves as a cofactor for CFI to cleave C3b to iC3b, alone or in the presence of CFH, while ULvWF lacks cofactor activity toward CFI or CFH and permits APC activation [21,22]. The concept that ULvWF binds C3b and acts as a platform for the assembly of C3/C5 convertase has been recently confirmed and expanded using plasma from a series of patients with congenital as well as acquired TTP [24].

Pecoraro et al. first described a 12-year-old boy with cTTP due to a compound heterozygous *ADAMTS13* mutation, who was successfully treated with eculizumab [11]. This patient, too, was initially misdiagnosed to have aHUS, and eculizumab induced prompt remission. Unlike our scenario, Pecoraro’s patient had severe acute kidney injury requiring dialysis. The authors also noted moderately increased plasma sC5b-9 concentrations. Renal function recovered quickly after anti-complement therapy, but TTP recurred with its discontinuation. Subsequent relapses reverted promptly with single doses of eculizumab. Pecoraro’s patient had no detectable complement factor mutations nor anti-CHF or anti-ADAMTS13 antibodies, albeit the number of genes tested was limited and did not include *CFHR3/1* [11].

In conclusion, complement activation, likely due to ULvWF-induced APC dysregulation, may contribute to the pathophysiology and clinical manifestations of TTP, especially hemolysis and likely, thrombocytopenia. The potential role of isolated CFHR3/1 deletion without detectable anti-CFH antibodies is incompletely understood. The present case and a previous report [11] suggest that anti-complement agents may have a role in the management of (some) patients with TTP, congenital, or acquired. While we are currently not advocating for complement blocking therapies in all TTP patients, we wish to highlight the merits of additional basic and clinical research beyond present guidelines. Different nosological entities as currently defined [25] may overlap, and comprehensive functional and genetic studies are needed to avoid diagnostic pitfalls, such as the presence of complement regulator mutations and/or anti-CFH antibodies [10,12,26], particularly in patients presenting with a complicated or protracted course of TTP. The pathophysiological role of dyregulated APC activation in TTP should be addressed in prospective studies.

## Abbreviations

ADAMTS13: A disintegrin and metalloprotease with a thromboSpondin type 1 motif, member 13
AH50: Alternative pathway of complement hemolytic activity
aHUS: atypical hemolytic uremic syndrome
APC: Alternative pathway of complement
aTTP: Acquired thrombotic thrombocytopenic purpura
CFH: Complement factor H
CFHR: Complement factor H-related protein
CFI: Complement factor I
CH50: Classical pathway of complement hemolytic activity
DEAP HUS: Deficiency of CFHR proteins and CFH autoantibody positive (anti-CFH antibody HUS)
cTTP: Congenital thrombotic thrombocytopenic purpura
ELISA: Enzyme-linked immune assay
FRETS-VWF73: Fluorescence resonance energy transfer assay with synthetic 73-aminoacid peptide
Hb: Hemoglobin
HUS: Hemolytic uremic syndrome
IgG: Immunoglobulin G
MAC: Membrane attack complex
PLEX: Plasma exchange
sMAC: Soluble (circulating) MAC
TMA: Thrombotic microangiopathy
TTP: Thrombotic thrombocytopenic purpura
ULvWF: Ultra-large vWF
vWF: von Willebrand factor

## References

[1] George JN, Nester CM (2014). Syndromes of thrombotic microangiopathy. N Engl J Med.

[2] Sadler JE (2017). Pathophysiology of thrombotic thrombocytopenic purpura. Blood.

[3] Lenting PJ, Christophe OD, Denis CV (2015). von Willebrand factor biosynthesis, secretion, and clearance: connecting the far ends. Blood.

[4] Joly BS, Coppo P, Veyradier A (2017). Thrombotic thrombocytopenic purpura. Blood.

[5] Vieira-Martins P, El Sissy C, Bordereau P, Gruber A, Rosain J, Fremeaux-Bacchi V (2016). Defining the genetics of thrombotic microangiopathies. Transfus Apher Sci.

[6] Jozsi M, Licht C, Strobel S, Zipfel SL, Richter H, Heinen S, Zipfel PF, Skerka C (2008). Factor H autoantibodies in atypical hemolytic uremic syndrome correlate with CFHR1/CFHR3 deficiency. Blood.

[7] Loirat C, Fakhouri F, Ariceta G, Besbas N, Bitzan M, Bjerre A, Coppo R, Emma F, Johnson S, Karpman D, Landau D, Langman CB, Lapeyraque AL, Licht C, Nester C, Pecoraro C, Riedl M, van de Kar NC, Van de Walle J, Vivarelli M, Fremeaux-Bacchi V, International HUS (2016). An international consensus approach to the management of atypical hemolytic uremic syndrome in children. Pediatr Nephrol.

[8] Greenbaum LA, Fila M, Ardissino G, Al-Akash SI, Evans J, Henning P, Lieberman KV, Maringhini S, Pape L, Rees L, van de Kar NC, Vande Walle J, Ogawa M, Bedrosian CL, Licht C (2016). Eculizumab is a safe and effective treatment in pediatric patients with atypical hemolytic uremic syndrome. Kidney Int.

[9] Kokame K, Nobe Y, Kokubo Y, Okayama A, Miyata T (2005). FRETS-VWF73, a first fluorogenic substrate for ADAMTS13 assay. Br J Haematol.

[10] Tsai E, Chapin J, Laurence JC, Tsai HM (2013). Use of eculizumab in the treatment of a case of refractory, ADAMTS13-deficient thrombotic thrombocytopenic purpura: additional data and clinical follow-up. Br J Haematol.

[11] Pecoraro C, Ferretti AV, Rurali E, Galbusera M, Noris M, Remuzzi G (2015). Treatment of congenital thrombotic thrombocytopenic Purpura with Eculizumab. Am J Kidney Dis.

[12] Sasapu A, Cottler-Fox M, Motwani P (2017). Acquired thrombotic thrombocytopenic purpura and atypical hemolytic uremic syndrome successfully treated with eculizumab. Proc (Bayl Univ Med Cent).

[13] Hageman GS, Hancox LS, Taiber AJ, Gehrs KM, Anderson DH, Johnson LV, Radeke MJ, Kavanagh D, Richards A, Atkinson J, Meri S, Bergeron J, Zernant J, Merriam J, Gold B, Allikmets R, Dean M, Group AMDCS (2006). Extended haplotypes in the complement factor H (CFH) and CFH-related (CFHR) family of genes protect against age-related macular degeneration: characterization, ethnic distribution and evolutionary implications. Ann Med.

[14] Dragon-Durey MA, Blanc C, Marliot F, Loirat C, Blouin J, Sautes-Fridman C, Fridman WH, Fremeaux-Bacchi V (2009). The high frequency of complement factor H related CFHR1 gene deletion is restricted to specific subgroups of patients with atypical haemolytic uraemic syndrome. J Med Genet.

[15] Noone D, Al-Matrafi J, Tinckam K, Zipfel PF, Herzenberg AM, Thorner PS, Pluthero FG, Kahr WH, Filler G, Hebert D, Harvey E, Licht C (2012). Antibody mediated rejection associated with complement factor h-related protein 3/1 deficiency successfully treated with eculizumab. Am J Transplant.

[16] Teoh CW, Riedl M, Licht C (2016). The alternative pathway of complement and the thrombotic microangiopathies. Transfus Apher Sci.

[17] Page EE, Kremer Hovinga JA, Terrell DR, Vesely SK, George JN (2016). Clinical importance of ADAMTS13 activity during remission in patients with acquired thrombotic thrombocytopenic purpura. Blood.

[18] Turner NA, Moake J (2013). Assembly and activation of alternative complement components on endothelial cell-anchored ultra-large von Willebrand factor links complement and hemostasis-thrombosis. PLoS One.

[19] Reti M, Farkas P, Csuka D, Razso K, Schlammadinger A, Udvardy ML, Madach K, Domjan G, Bereczki C, Reusz GS, Szabo AJ, Prohaszka Z (2012). Complement activation in thrombotic thrombocytopenic purpura. J Thromb Haemost.

[20] Tati R, Kristoffersson AC, Stahl AL, Rebetz J, Wang L, Licht C, Motto D, Karpman D (2013). Complement activation associated with ADAMTS13 deficiency in human and murine thrombotic microangiopathy. J Immunol.

[21] Rayes J, Roumenina LT, Dimitrov JD, Repesse Y, Ing M, Christophe O, Jokiranta TS, Halbwachs-Mecarelli L, Borel-Derlon A, Kaveri SV, Fremeaux-Bacchi V, Lacroix-Desmazes S (2014). The interaction between factor H and VWF increases factor H cofactor activity and regulates VWF prothrombotic status. Blood.

[22] Feng S, Liang X, Kroll MH, Chung DW, Afshar-Kharghan V (2015). von Willebrand factor is a cofactor in complement regulation. Blood.

[23] Noone DG, Riedl M, Pluthero FG, Bowman ML, Liszewski MK, Lu L, Quan Y, Balgobin S, Schneppenheim R, Schneppenheim S, Budde U, James P, Atkinson JP, Palaniyar N, Kahr WH, Licht C (2016). Von Willebrand factor regulates complement on endothelial cells. Kidney Int.

[24] Bettoni S, Galbusera M, Gastoldi S, Donadelli R, Tentori C, Sparta G, Bresin E, Mele C, Alberti M, Tortajada A, Yebenes H, Remuzzi G, Noris M (2017). Interaction between multimeric von Willebrand factor and complement: a fresh look to the pathophysiology of microvascular thrombosis. J Immunol.

[25] Cataland SR, Holers VM, Geyer S, Yang S, Wu HM (2014). Biomarkers of terminal complement activation confirm the diagnosis of aHUS and differentiate aHUS from TTP. Blood.

[26] Chapin J, Weksler B, Magro C, Laurence J (2012). Eculizumab in the treatment of refractory idiopathic thrombotic thrombocytopenic purpura. Br J Haematol.

